# Expanding the Mutational Spectrum of TSPEAR in Ectodermal Dysplasia Type 14: A Familial Case Study

**DOI:** 10.3390/genes16050519

**Published:** 2025-04-29

**Authors:** Roberto Sirica, Alessandro Ottaiano, Daniele De Brasi, Simone Marcella, Fabio Acquaviva, Monica Ianniello, Nadia Petrillo, Valentina De Angelis, Raffaella Ruggiero, Rossana D’Angelo, Eloisa Evangelista, Antonio Fico, Giovanni Savarese

**Affiliations:** 1 AMES, Centro Polidiagnostico Strumentale srl, Via Padre Carmine Fico 24, 80013 Casalnuovo di Napoli, Italy; roberto.sirica@centroames.it (R.S.); simone.marcella@centroames.it (S.M.); monica.ianniello@centroames.it (M.I.); nadia.petrillo@centroames.it (N.P.); raffaella.ruggiero@centroames.it (R.R.); rossana.dangelo@centroames.it (R.D.); eloisa.evangelista@centroames.it (E.E.); antonio.fico@centroames.it (A.F.); 2 Istituto Nazionale Tumori di Napoli, IRCCS “G. Pascale”, Via Mariano Semmola, 80131 Napoli, Italy; a.ottaiano@istitutotumori.na.it; 3 Azienda Ospedaliera di Rilievo Nazionale, “Santobono Pausilipon”, Via Teresa Ravaschieri 8, 80122 Napoli, Italy; d.debrasi@santobonopausillipon.it (D.D.B.); f.acquaviva@santobonopausillipon.it (F.A.); 4 Istituto Comprensivo Statale “Massimo Troisi”, Via Pini di Solimena 31, 80046 San Giorgio a Cremano, Italy; val.angelis@libero.it

**Keywords:** ectodermal dysplasia, next-generation sequencing, *TSPEAR*, oligodontia, genotype–phenotype correlation

## Abstract

Background: Ectodermal dysplasia (ED) encompasses a heterogeneous group of genetic disorders affecting ectoderm-derived structures such as hair, teeth, nails, and sweat glands. Among these, variants in *TSPEAR* (Thrombospondin-type laminin G domain and epilepsy-associated repeats) have been implicated in autosomal recessive ED type 14 (OMIM 618180), predominantly manifesting with dental anomalies and hair dysplasia. However, the mutational spectrum of *TSPEAR* remains incompletely characterized. Methods: Two female siblings (ID#1 and ID#4) were clinically evaluated for ED. Genetic analysis, including next-generation sequencing (NGS) and Sanger validation, was conducted to identify *TSPEAR* variants. A segregation study confirmed inheritance patterns within the family. Results: Both affected siblings exhibited hallmark features of *TSPEAR*-related ED14, including oligodontia with dysmorphic, pointed maxillary central incisors. Hair thinning and cutaneous angiomas were predominant in ID#4. Genetic analysis identified two compound heterozygous variants in *TSPEAR*: c.543-1G>A, a splice-site variant likely to disrupt mRNA processing, and NM_144991.2:c.1251G>C(p.Gln417His), a missense variant with predicted deleterious effects. Segregation analysis confirmed maternal and paternal inheritance of the respective variants. A third sibling, ID#5, was identified as a heterozygous carrier without clinical manifestations. Conclusions: This study contributes to the expanding understanding of *TSPEAR*-related ED14 by providing novel genotype–phenotype correlations.

## 1. Introduction

Ectodermal dysplasia (ED) comprises a heterogeneous group of genetic disorders affecting the development of ectoderm-derived structures, including hair, teeth, nails, and sweat glands [1]. One of the most well-characterized forms is hypohidrotic ectodermal dysplasia (HED), typically caused by variants in *EDA* (ectodysplasin A), *EDAR* (ectodysplasin A receptor), or *EDARADD* (EDAR-associated via death domain), and is defined by hypohidrosis, oligodontia with conical teeth, and sparse hair [2]. Anhidrotic ectodermal dysplasia (AED) involves the same genetic alterations but represents a more severe variant, characterized by a near-complete absence of sweating, leading to significant thermoregulatory challenges [3,4]. ED can be associated with pathogenic variants in *WNT10A* (Wingless-Type MMTV Integration Site Family, Member 10A), typically presenting with dental anomalies such as hypodontia or anodontia and dysmorphic, conical maxillary central incisors. The phenotypic spectrum of *WNT10A*-related ED is broad, ranging from isolated mild dental abnormalities to more severe manifestations resembling HED [5]. Beyond these well-established genes, *TSPEAR* (Thrombospondin-Type Laminin G Domain and EAR Repeats) has emerged as a relevant candidate, particularly in autosomal recessive forms of ED (ED type 14; OMIM 618180), which are predominantly characterized by dental and hair anomalies [1].

Pathogenic variants in *TSPEAR* have been associated with a phenotype characterized by oligodontia, nail dystrophy, and sparse hair, often accompanied by additional dermatological manifestations, reduced sweating, and craniofacial abnormalities. However, no direct correlation with intellectual or cognitive impairment has been reported [1,6]. The exact incidence of *TSPEAR*-related ED in Italy and Europe remains undefined due to the rarity of this condition and the broader classification of ED under different genetic subtypes. However, ED as a group hasan estimated prevalence of 1 in 10,000 to 1 in 100,000 live births [1,2,4]. However, the carrier frequency of *TSPEAR* variants in the non-Finnish European population is approximately 1 in 140. This suggests that ED type 14 may be among the most prevalent autosomal recessive ectodermal dysplasias [1]. The *TSPEAR* gene is located on chromosome 21q22.3 and encodes a protein thought to be involved in extracellular matrix organization and cellular interactions essential for ectodermal development. While its precise molecular function remains incompletely characterized, it has been suggested that TSPEAR plays a role in modulating Notch signaling, a pathway critical for epithelial and mesenchymal cell differentiation during organogenesis [7]. This functional context aligns with the observed phenotypic alterations in patients harboring *TSPEAR* variants, particularly in structures derived from ectodermal–mesenchymal interactions. Pathogenic variants in *TSPEAR* typically include frameshift and nonsense variants leading to premature truncation of the protein, as well as splice-site alterations that disrupt normal transcript processing and missense variants with high allele frequencies, including p.G475S and p.G498V in EAR4, p.Y566C in EAR5, p.S585I in EAR6, and p.D639N in EAR7 [1,8,9]. However, the genotype–phenotype correlation in *TSPEAR*-related ED remains an area of ongoing investigation. Variability in clinical severity among individuals carrying the same variant suggests the presence of modifier genes or epigenetic influences affecting phenotypic expression. Moreover, incomplete penetrance and variable expressivity complicate the clinical diagnosis, emphasizing the necessity of molecular genetic testing for confirmation.

Here, we report the case of two sisters affected by ED type 14, which was subsequently confirmed through a next-generation sequencing (NGS) study.

## 2. Materials and Methods

### 2.1. Subjects

The cases ID#1 and ID#4 were clinically and phenotypically evaluated by an expert clinical geneticist (R.R.) who proposed a clinical diagnosis of ED. The parents gave informed consent for genetic assessments and article writing and publication. This study was approved by the IRB of the Centro AMES with protocol. n. CA02/2025.

### 2.2. DNA Sequencing and Variant Validation

Genomic DNA (gDNA) was isolated from peripheral blood collected in EDTA tubes, following the protocol provided by the manufacturer (MagCore Nucleic Acid Extraction Kit, Diatech Pharmacogenetics, Jesi, Italy). For amniotic fluid samples, gDNA was extracted from cultured amniocytes using the QIAamp DNA Blood Mini Kit (Qiagen, Hilden, Germany). DNA concentrations were determined with a Qubit 3.0 Fluorometer employing the Qubit dsDNA High Sensitivity Assay Kit. DNA obtained was used for library preparation and probe enrichment with Kapa Hyper Plus Exome Probes according to the manufacturer’s protocol (Kapa Biosystems, Roche Diagnostics, Wilmington, MA, USA).

Sequencing was carried out on NovaSeq 6000 (Illumina Inc., San Diego, CA, USA) to mean sequencing depth of at least 100X. Sequence data were aligned to the human reference genome GRCh37 (http://www.ncbi.nlm.nih.gov/projects/genome/assembly/grc/human/index.shtml, accessed on 1 February 2018) using the Burrows–Wheeler Aligner with default parameters. Trimming, base calling, coverage analysis, and variant calling were performed using an in-house bioinformatic pipeline using free software, respectively, as follows: bcl to fastq version 2.20, Isaac Aligner version 4, GATK “Genome Analysis Toolkit” version 4, samtools version 1.9, and bedtools version 2.

VCF analysis was conducted using the Illumina Variant Interpreter, applying filters for quality >15 and focusing on small variant effects, such as stop codon gains, losses, splice donor and acceptor sites, splice regions, frameshift indels, in-frame insertions and deletions, initiator codon (ATG) deletions, missense mutations, and incomplete terminal codons.

To confirm the variant, Sanger sequencing was performed on the buccal swab sample and on heterozygous individuals through targeted sequencing. Primers were designed using Primer3 (http://bioinfo.ut.ee/primer3-0.4.0/, accessed on 15 February 2018), ensuring they were located at least 100 bp upstream and downstream of the variant. Primer sequences can be provided upon request.

## 3. Case Report

**Figure 1:**
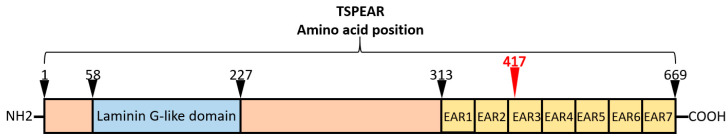
Schematic representation of the TSPEAR (Thrombospondin-type laminin G domain and epilepsy-associated repeats). The protein corresponds to transcript NM_144991.2 and protein NP_659428.2. The position of the variant inherited from the father is indicated in red (TSPEAR NM_144991.2:c.1251G>C; p.Gln417His). The variant inherited from the mother affects an intronic splice acceptor site (c.543-1G>A).

ID#1 is a female child born at term in 2020, conceived naturally after ultrasound follow-up, with no detected anomalies. Apgar scores and otoacoustic emissions (OAEs) were within the normal range. At the age of two, she received a clinical diagnosis of ED type 14 (OMIM 618180), which was subsequently confirmed through next-generation sequencing (NGS). Genetic analysis revealed two compound heterozygous variants in the *TSPEAR* gene: c.543-1G>A, a splice-site variant likely to disrupt mRNA processing, and a missense variant, NM_144991.2:c.1251G>C(p.Gln417His) (Figure 1).

Consequently, in late May 2022, a segregation study was conducted on both parents (mother: ID#2; father: ID#3) using NGS to investigate the presence of the same variants detected in their daughter. The study confirmed that one variant was maternally inherited and the other was of paternal origin. In March 2023, a second female child (ID#4) was born at term, conceived naturally, with no detected anomalies during prenatal ultrasound follow-up. Apgar scores and OAEs were normal. However, prenatal genetic testing via amniocentesis for familial *TSPEAR* variants revealed the presence of both pathogenic variants, confirming a compound heterozygous state, predictive of ED type 14.

In December 2024, the couple’s third child, a male, was born following an uneventful full-term pregnancy and spontaneous delivery. Prenatal ultrasound follow-up showed no abnormalities, and Apgar scores and OAEs were normal. GDNA analysis from a buccal swab demonstrated that the child had inherited only the paternally derived variant, indicating a carrier status similar to that of the father. Anonymized sequence data of ID#1, 2, 3, and 4 have been deposited in the European Nucleotide Archive (ENA) (Supplementary File S1). The molecular report of the most recently born child is provided in Supplementary File S2.

**Figure 2:**
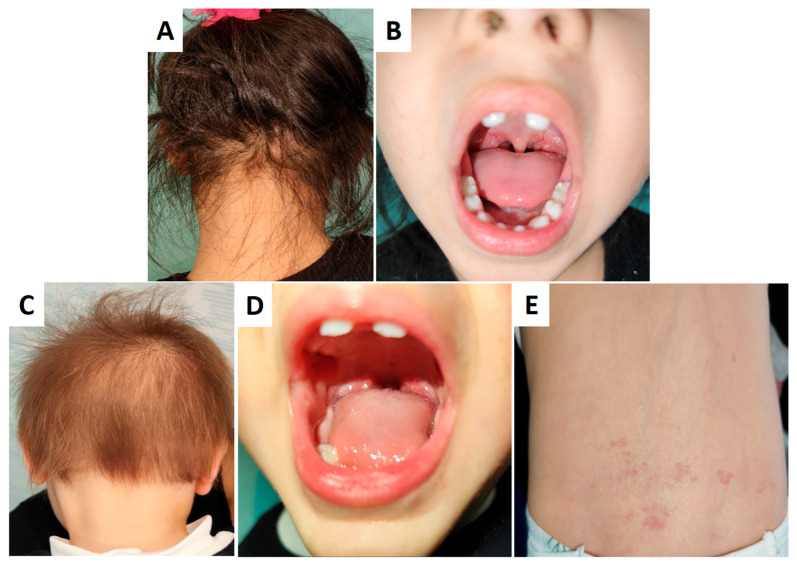
Phenotypic manifestations of cases ID#1 (**A**,**B**) and ID#4 (**C**–**E**). Both ID#1 and ID#4 exhibit dysmorphic, pointed maxillary central incisors (**B**,**D**). ID#4 shows more pronounced cutaneous involvement, including increased hair thinning (**C**) compared to ID#1 (**A**) and angiomas in the lumbosacral region (**E**).

Both ID#1 and ID#4 exhibited the eruption of dysmorphic, pointed maxillary central incisors at 13 months of age. ID#4 showed more pronounced cutaneous involvement, with increased hair thinning and angiomas in the lumbosacral area (Figure 2). Sweating, nail morphology, and tear production were within normal limits. No signs of neuropathic pain or reflex abnormalities were detected. Intellectual and cognitive development and overall growth were normal.

## 4. Discussion

The present case report describes two siblings affected by ED type 14 due to compound heterozygous variants in *TSPEAR*, alongside a third sibling identified as a healthy carrier. This study contributes to the expanding knowledge of *TSPEAR*-related ED by providing detailed phenotypic characterization and genetic evidence supporting the pathogenicity of these variants. The first variant, c.543-1G>A, is a heterozygous splice acceptor site variant not reported in the ClinVar database but listed in dbSNP (rs1182814378) as a variant of unknown clinical significance. SpliceAI analysis (https://spliceailookup.broadinstitute.org/, last accessed on 21 April 2025) predicts loss of the acceptor splice site with a high probability score (0.99). According to the ACMG classification criteria (Supplementary File S3), this variant is considered likely pathogenic (PVS1, PM2) (http://varso.me/c4iT, accessed on 21 April 2025). The second variant, c.1251G>C, resulting in the p.Gln417His substitution, is also present in heterozygosity. It is not reported in ClinVar but is listed in dbSNP (rs1555914851) as a variant of unknown clinical significance. Based on ACMG criteria, this variant is classified as a variant of uncertain significance (VUS) (PM2, PP3) (http://varso.me/c4j5, accessed on 21 April 2025).

TSPEAR has been implicated in ED due to its role in extracellular matrix interactions and cell signaling during ectodermal development [4]. In this report, affected individuals (ID#1 and ID#4) exhibited hallmark features of *TSPEAR*-related ED; however, other ectodermal structures, such as sweat glands, nails, and lacrimal glands, remained unaffected.

From a genetic perspective, the two compound heterozygous variants identified in *TSPEAR* contribute to the current understanding of pathogenic mechanisms underlying this condition. The c.543-1G>A variant, located at a canonical splice acceptor site, is highly likely to disrupt mRNA processing, leading to aberrant splicing and probable loss of function. This aligns with previous reports that describe truncating or splicing variants as disease-causing in *TSPEAR*-related ED [9]. The second variant, c.1251G>C (p.Gln417His), represents a missense alteration with an uncertain clinical significance according to ACMG guidelines (PM2, PP3). However, in the context of a second truncating variant, its contribution to disease pathogenesis cannot be excluded, especially given the evolutionary conservation of the affected amino acid. Further functional studies are warranted to elucidate its impact on protein function.

The phenotypic presentation in our cases underscores the variability observed in ED. Although both affected siblings carried the same genotype, ID#4 exhibited more pronounced cutaneous involvement, with increased hair thinning and angiomas. This suggests potential modifier factors, either genetic or environmental, influencing disease severity. The role of epigenetic regulation and interactions with other signaling pathways, such as Notch, Wnt, and BMP, could modulate the phenotypic spectrum. Additionally, incomplete penetrance has been noted in other ED subtypes, raising the possibility of additional regulatory elements contributing to individual expression of clinical features [9].

The identification of compound heterozygous variants in *TSPEAR* has significant implications for genetic counseling, particularly regarding recurrence risk and prenatal diagnosis. The couple in this study underwent targeted testing for familial variants in subsequent pregnancies, allowing for early diagnosis via amniocentesis. The confirmed carrier status of their third child emphasizes the importance of molecular testing in family planning, as heterozygous carriers remain asymptomatic but can transmit pathogenic alleles to future offspring. The absence of evident intellectual or cognitive impairment in affected individuals further supports the notion that *TSPEAR*-related ED primarily affects ectodermal structures, distinguishing it from syndromic forms with neurodevelopmental involvement.

While this study provides valuable insights into *TSPEAR*-related ED, several limitations should be acknowledged. Functional validation of the identified variants was not performed, leaving their precise molecular consequences unconfirmed. RNA studies or in vitro assays would be necessary to establish the exact impact of the splice-site variant on transcript processing. Furthermore, long-term follow-up of affected individuals could provide additional data on disease progression, particularly regarding dental anomalies and potential late-onset symptoms.

Given the nature of the dental abnormalities observed in this form of ED, long-term orthodontic follow-up is essential to monitor the development of dentition and determine the need for corrective interventions, such as prosthetic rehabilitation or orthodontic alignment. Additionally, dermatological surveillance remains crucial, particularly for patients with significant cutaneous involvement, to assess potential complications such as skin fragility or increased susceptibility to infections. Equally important is the psychological follow-up of affected individuals, considering the potential impact of self-image distortion on social interactions and emotional well-being. Children with visible dental and dermatological abnormalities may experience self-esteem challenges and social stigma, highlighting the necessity of early psychological support and counseling to mitigate long-term psychosocial consequences [10].

This case report highlights the pathogenic role of novel *TSPEAR* variants in ED type 14, expanding the mutational spectrum associated with this condition. The observed phenotypic variability suggests the influence of genetic and epigenetic modifiers, warranting further investigation. Our findings underscore the importance of molecular diagnosis in ED for accurate genetic counseling and early detection in at-risk pregnancies. Future studies should focus on elucidating the functional consequences of *TSPEAR* variants and their broader implications in ectodermal development.

## Data Availability

The original data presented in this study are publicly available in the European Nucleotide Archive (ENA) (see Supplementary File S1) and as an original molecular report (Supplementary File S2).

## Supplementary Materials

The following supporting information can be downloaded at https://www.mdpi.com/article/10.3390/genes16050519/s1: Supplementary File S1: Deposition of NGS sequences in ENA ID#1, 2, 3, 4. Supplementary File S2: Molecular report Sanger sequence ID#5. Supplementary File S3: ACMG criteria for the classification of genetic variants. Reference [11] is cited in the Supplementary Materials.
