# Supplementary material for: Expanding the Mutational Spectrum of TSPEAR in Ectodermal Dysplasia Type 14: A Familial Case Study
Source: Genes (Basel). 2025 Apr 29;16(5):519. doi: 10.3390/genes16050519 (PMC12111227; doi:10.3390/genes16050519)
Supplement: Supplementary file 1 [file genes-16-00519-s001.zip › genes-3578110-supplementary/Supplementary File S1.pdf]

Supplementary File S1

Deposition of NGS sequences in ENA ID#1, 2, 3, 4

The clinical case is detailed in the study "Expanding the Mutational Spectrum of TSPEAR in Ectodermal Dysplasia Type 14: A Familial Case Study."

Patient sequence data have been deposited in the European Nucleotide Archive (ENA): https://www.ebi.ac.uk/ena/browser/home last accessed on 11 February 2025.

ENA RUN IDs:

ID#1:
ERS23821387 (AM-SP1) – Sample origin: Blood (First daughter)

ID#2:
ERS23821389 (AM-SP3) – Sample origin: Blood (Mother)

ID#3:
ERS23821388 (AM-SP2) – Sample origin: Blood (Father)

ID#4:
ERS23821390 (AM-SP4) – Sample origin: Amniotic fluid (Second daughter)
