# Supplementary material for: Expanding the Mutational Spectrum of TSPEAR in Ectodermal Dysplasia Type 14: A Familial Case Study
Source: Genes (Basel). 2025 Apr 29;16(5):519. doi: 10.3390/genes16050519 (PMC12111227; doi:10.3390/genes16050519)
Supplement: Supplementary file 1 [file genes-16-00519-s001.zip › genes-3578110-supplementary/Supplementary File S2.pdf]

Supplementary File S2

[redacted]

Acc.nro:33678 del 31/01/2025

REFERTO DI ANALISI MOLECOLARE SU DNA
RICERCA DI VARIANTI SPECIFICHE NEI GENI TSPEAR E PKP1

RISULTATI

GENE ID	VARIANTE GENICA RICERCATA	RISULTATO
TSPEAR (NM_144991.2)	c.543-1G>A	**Variante Assente**
TSPEAR (NM_144991.2)	c.1251G>C (p.Gln417His)	**Variante Presente**

L'analisi molecolare eseguita sul DNA estratto da tampone buccale del paziente in esame, utilizzando le metodiche di seguito descritte e per quello che le stesse possono rivelare a livello genico, **ha evidenziato** le seguenti varianti:
- **c.1251G>C** (p.Gln417His) in eterozigosi nel gene TSPEAR

I risultati di tale diagnosi molecolare vanno interpretati dal Medico richiedente e/o dal Medico Genetista tenendo conto degli altri risultati di laboratorio, del quadro clinico e dei dati anamnestici del paziente.

METODICHE UTILIZZATE

- Estrazione del DNA (MagCore Nucleic Acid Extraction Kit, diatech pharmacogenetics).
- PCR (Polymerase Chain Reaction): amplificazione del DNA mediante PCR per l'analisi della regione genica di interesse (AmpliTaq Gold® 360 DNA Polymerase, Applied Biosystems by life technologies).
- Sequenziamento diretto con il metodo di Sanger: gli amplificati ottenuti sono separati mediante elettroforesi capillare (ABI-PRISM 3500, Applied Biosystems) ed analizzati mediante Sequencing Analysis versione 6 (Applied Biosystems).
- Limiti del test: non si può escludere la presenza di rare varianti geniche, nelle regioni di annealing dei primers, che possano interferire con l'analisi molecolare.

Controllo di qualità: Il percorso analitico prevede l'analisi di un bianco campione per serie analitica per verificare l'assenza di contaminazioni da DNA esogeno (controllo negativo).
L'azienda è certificata secondo la normative di riferimento UNI EN ISO 9001:2015 e **SIGUCERT** per i settori di **Genetica Molecolare, Citogenetica** e **Farmacogenetica**.

Responsabile del Centro Ames	***Dott.ssa Rossana D'Angelo***	***Dott.ssa Raffaella Ruggiero***
Dr. Antonio Fico	***Responsabile fase analitica***	***Coordinatore Settore di Genetica***

<u>**We provide a translation of the final result from Italian to English (as shown in the yellow rectangle).**</u>

The molecular analysis performed on DNA extracted from the patient's buccal swab, using the methods described below and within the detectable limits of these techniques at the gene level, identified the following variant:

c.1251G>C (p.Gln417His) in heterozygosity in the TSPEAR gene.
