# Supplementary material for: Expanding the Mutational Spectrum of TSPEAR in Ectodermal Dysplasia Type 14: A Familial Case Study
Source: Genes (Basel). 2025 Apr 29;16(5):519. doi: 10.3390/genes16050519 (PMC12111227; doi:10.3390/genes16050519)
Supplement: Supplementary file 1 [file genes-16-00519-s001.zip › genes-3578110-supplementary/Supplementary File S3.pdf]

Pathogenic Criteria of ACMG (American College of Medical Genetics):

Very Strong (PVS1):
Null variant (nonsense, frameshift, canonical ±1 or 2 splice sites, initiation codon, or single/multiexon deletion) in a gene where loss of function is a known mechanism of disease.

Strong (PS1–PS4):
PS1: Same amino acid change as a previously established pathogenic variant (but different nucleotide change).
PS3: Well-established functional studies show a damaging effect.
PS4: Prevalence of the variant in affected individuals is significantly increased compared to controls.

Moderate (PM1–PM6):
PM1: Located in a mutational hot spot or critical domain.
PM2: Absent (or very rare) in population databases (e.g., gnomAD).
PM5: Novel missense change at a residue where another pathogenic missense change has been observed.

Supporting (PP1–PP5):
PP1: Co-segregation with disease in multiple affected family members.
PP3: Multiple computational predictions support a deleterious effect (e.g., SpliceAI, SIFT, PolyPhen-2).
PP4: Patient's phenotype or family history is highly specific for a disease with a single genetic cause.

Combining Criteria – Classification Rules:

Classification	Minimum Required Criteria
Pathogenic	1 Very Strong + ≥1 Strong OR 2 Strong + ≥2 Moderate OR 1 Strong + ≥3 Moderate + ≥2 Supporting
Likely Pathogenic	1 Very Strong + 1 Moderate OR 1 Strong + 1–2 Moderate OR ≥3 Moderate
Benign	1 Stand-alone OR ≥2 Strong benign evidence
Likely Benign	1 Strong + 1 Supporting OR ≥2 Supporting
VUS (Uncertain)	Conflicting evidence or insufficient support for any other classification

References

Richards S, et al. Standards and guidelines for the interpretation of sequence variants: a joint consensus recommendation of the American College of Medical Genetics and Genomics and the Association for Molecular Pathology. Genet Med. 2015 May;17(5):405-24. doi: 10.1038/gim.2015.30.
